# Learning to use vestibular sense for spatial updating is context dependent

**DOI:** 10.1038/s41598-019-47675-7

**Published:** 2019-08-01

**Authors:** Isabelle Mackrous, Jérôme Carriot, Martin Simoneau

**Affiliations:** 1 Department of Physiology, McGill University, Montreal, QC Canada; 2 Centre Interdisciplinaire de Recherche en Réadaptation et Intégration Sociale (CIRRIS), Québec, QC Canada; 3 Département de kinésiologie, Faculté de médecine, Université Laval, Québec, QC Canada

**Keywords:** Sensory processing, Sensorimotor processing

## Abstract

As we move, perceptual stability is crucial to successfully interact with our environment. Notably, the brain must update the locations of objects in space using extra-retinal signals. The vestibular system is a strong candidate as a source of information for spatial updating as it senses head motion. The ability to use this cue is not innate but must be learned. To date, the mechanisms of vestibular spatial updating generalization are unknown or at least controversial. In this paper we examine generalization patterns within and between different conditions of vestibular spatial updating. Participants were asked to update the position of a remembered target following (offline) or during (online) passive body rotation. After being trained on a single spatial target position within a given task, we tested generalization of performance for different spatial targets and an unpracticed spatial updating task. The results demonstrated different patterns of generalization across the workspace depending on the task. Further, no transfer was observed from the practiced to the unpracticed task. We found that the type of mechanism involved during learning governs generalization. These findings provide new knowledge about how the brain uses vestibular information to preserve its spatial updating ability.

## Introduction

One remarkable achievement of the brain is its ability to preserve a stable perception of the environment as we move. To maintain perceptual stability, it is vital that the brain continually accounts for self-motion to keep track of object positions around us. The prevailing view, known as the spatial updating mechanism, is that the brain updates the retinotopic map during motion to preserve spatial constancy^1,2^. Sensory cues and efferent copy signals are used as the sources of updating information^3^. As everyday activities involve head and body movements, information about self-motion should also be incorporated when updating the visual field. Because the vestibular system encodes head motion (see^4^), it has been proposed that it contributes to spatial updating^3,5–9^.

The mechanism that processes vestibular inputs to update the retinotopic map during head movement is not fully understood. Nonetheless, there are several reasons to believe that learning is involved. On one hand, previous experiments have shown that the brain fails to accurately reconstruct the position of a visual target following passive body rotation in darkness^5,10^. On the other hand, the ability to use vestibular inputs to perform spatial updating has been observed when participants are given feedback about their spatial accuracy^7,11,12^. The recovery of performance when participants learn to use vestibular inputs suggests that the brain can maintain spatial accuracy by changing its sensory cues to update spatial information. In this regard, numerous psychophysical and neurophysiological studies have shown that the process of changing sensory cues is a dynamic process implemented over different time scales to maintain a functional level of performance when facing an impoverished sensory context^13–18^. Importantly, studies have also shown that this process is task-specific^15,19–22^. Nevertheless, the ability of the brain to learn to use vestibular input to maintain optimized spatial updating performance was robustly found in different contexts of a spatial updating task^7,8,11,12^ (i.e., when spatial information needed to be updated during or following motion) using different types of responses. To date, however, it remains unknown whether this finding represents similar learning mechanisms when vestibular spatial updating occurs following or during body motion.

Therefore, we tested generalization patterns within and between different dynamic conditions of vestibular spatial updating. If similar learning mechanisms are involved when updating visual space during and after body motion, we should expect some generalization between the tasks. For instance, to update the representation of the visual field, vestibular velocity signals must be integrated into a positional signal^3,5,7,8,12^. This integration is mandatory either during or after body motion and should promote generalization. However, vestibular integration could be processed differently regardless of whether the updating of the visual space needs to be realized during (online) or after (offline) body motion. Such conditions put different time constraints on the sensory integration mechanisms, which could limit generalization of vestibular spatial updating. Thus, we investigated what controls generalization during vestibular spatial updating.

To evaluate generalization within a given task, we tested transfer of performance to different spatial targets. After training (i.e., online spatial updating or offline spatial updating), generalization was observed as performance transferred between the trained spatial targets and the untrained spatial targets. However, we also tested generalization of performance from one task to the other. Surprisingly, no generalization between the tasks was observed. These results shed new light on the processes that the brain undertakes when changing its sensory inputs in general, and on the brain’s use of vestibular signals to perform spatial updating.

## Results

### Offline processing

**Figure 1:**
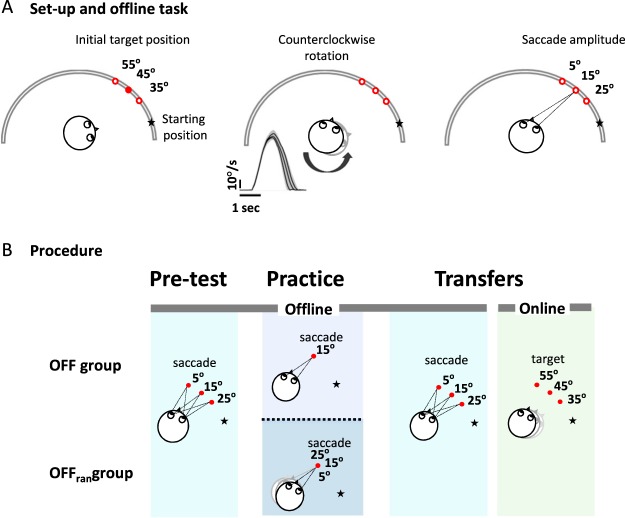
(**A**) Set-up. Participants sat on a rotating chair facing a semicircular panel. Targets were located at 35°, 45° or 55° in the counter-clockwise direction from the participant’s midline which faced the starting position at the beginning of a trial (left panel). For each trial, participants were rotated counter-clockwise (middle panel). Participants were rotated at three different chair velocities. Shaded areas represent the standard deviation (inset). For the OFF groups, participants were asked to gaze at the remembered target position following the rotation (right panel). (**B**) Offline procedure description: The star shows the starting position before rotation and the red dots illustrate the positions of the initial targets. Pre-test: Once the chair was stopped at its final position, the subject had to produce a saccade to the memorized/updated position of the initial target corresponding to saccade responses of either 25°, 15° or 5° respectively. Practice: OFF group: In every trial, a 15° saccade response had to be produced to gaze accurately at the memorized/updated position of the initial target. OFF_ran_: the amplitude of the required saccade response was varied pseudo-randomly across trials. To gaze accurately at the target, the participant had to produce either a 25°, 15° or 5° saccade response. Transfers: Participants reproduced the task they performed during the pre-test (offline task, shaded blue box). Thereafter, participants performed the transfer in the online condition (shaded green box).

In our everyday life, object positions can be updated following body motion. Hereafter, we refer to this process as offline spatial updating. During this task, initial targets were presented to the left of each participant’s midline before body rotation (Fig. 1A left panel). Following a passive body rotation (Fig. 1A middle panel), participants had to produce a saccade toward the new target’s position relative to the participant’s midline (Fig. 1A right panel). To evaluate whether offline spatial updating is generalized, we compared the performance of two groups, OFF and OFF_ran_, in pre-test, practice and transfer conditions (Fig. 1B).

### Pre-test condition: offline task

**Figure 2:**
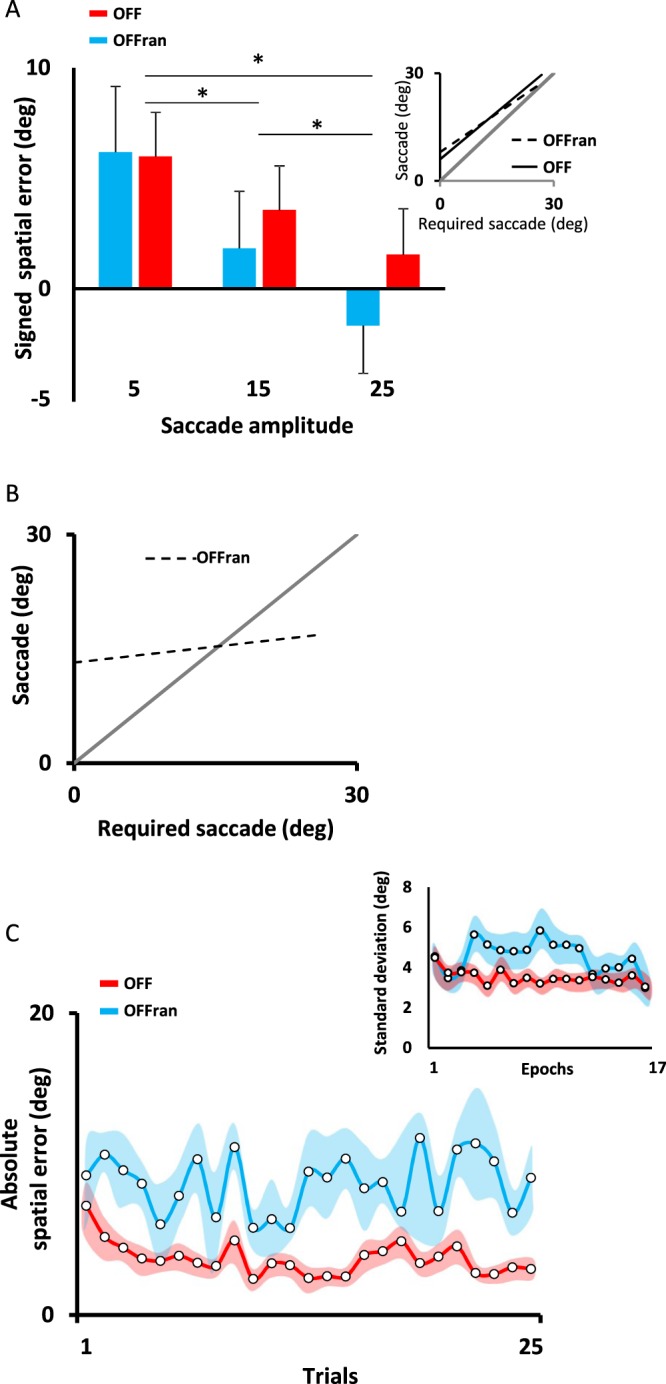
(**A**) Pre-test offline. Signed spatial error as a function of the required saccade amplitude. Signed spatial errors decreased as the required saccade amplitude increased. Error bars represent the standard error and * represents a p < 0.05. *Inset*. Mean regression lines for the OFF_ran_ (dashed line) and the OFF (black line) groups. For clarity, individual data are not shown. (**B**) Mean regression line for the OFF_ran_ (dashed line) groups during practice. Slope of the regression was reduced compared to the identity line (gray line), showing large anisotropy in the saccade response. (**C**) Absolute spatial error. Participants from the OFF_ran_ group did not show any decrease in their spatial error during practice (cyan trace). Slight but significant decrease of the spatial error was observed for the OFF group (red trace). Shaded areas represent the standard error. *Inset*. Participants from the OFF_ran_ group showed greater inter-trial variability in their saccade amplitude than the OFF group.

During pre-test, the initial targets were presented at 55°, 45° or 35° to the left of each participant’s midline before body rotation. Following a 60° passive body rotation, participants had to produce a saccade with an amplitude of 5° ± 0.8°, 15° ± 0.7° or 25° ± 0.8° (mean ± SD) to gaze accurately at the new target’s position relative to the participant’s midline. As shown in Fig. 2A (*inset*), a significant correlation was found between the actual saccade and the saccade amplitude required to gaze at the target. The averaged regression lines had a slope of 0.88 ± 0.06 (R^2^ = 0.70) and 0.72 ± 0.09 (R^2^ = 0.52) and an intercept of 6° ± 1.8° and 8° ± 3.2° (mean ± SE) for the OFF and OFF_ran_ groups, respectively. No significant difference was found between groups for the slope (one-way ANOVA, *F*_(2, 18)_ = 2.7, *p* = 0.12) and the intercept (one-way ANOVA, *F*_(2, 18)_ = 0.5, *p* = 0.51). Nonetheless, participants tended to slightly overshoot the position of the targets and the signed spatial error varied with the saccade amplitude (Fig. 2A). As the amplitude of the saccade increased, the signed spatial error decreased (main effect of saccade amplitude: *F*_(2, 40)_ = 18.6, *p* < 0.001).

### Practice condition: offline task

To evaluate performance in the presence of external feedback, participants were trained to update the position of a memorized target that was located at 45° to the left of their midline. Following a passive body rotation of 60° amplitude, participants from the OFF group were required to produce a saccade amplitude of 15° ± 0.7° (mean ± SD) to accurately gaze at the memorized target. For the OFF_ran_ group, rotation amplitude varied pseudo-randomly across trials (i.e., 50°, 60° or 70°). Accordingly, the OFF_ran_ group had to produce saccade amplitudes of 5° ± 0.7°, 15° ± 0.7° or 25° ± 0.7° (mean ± SD) to accurately gaze at the memorized target. External feedback about their spatial error was given after each trial. As illustrated in Fig. 2B (dashed line), the OFF_ran_ group showed poor scaling of the actual saccade amplitude with the required saccade amplitude. Averaged across participants, the regression line had a slope of 0.14 ± 0.09 and an intercept of 13.2° ± 6.6° (mean ± SD), R^2^ = 0.10. The small slope was caused by the large anisotropy in the spatial error observed between saccade amplitudes. For instance, when the required saccade was of a small amplitude, participants tended to overshoot the position of the target, while the opposite was observed for large saccade amplitudes. Figure 2C illustrates the absolute spatial error across practice trials. For the OFF_ran_ group, practice did not improve performance, as shown by the lack of decrease for the absolute spatial error (blue traces). Conversely, absolute spatial error slightly decreased within the first 10 trials for the OFF group (red traces). The divergent patterns of improvement for the OFF_ran_ and OFF groups are supported by a significant interaction [Group (OFF and OFF_ran_) x Epochs (3 first trials vs. 3 last trials), ANOVA *F*_(1, 20)_ = 5.9, *p* = 0.024]. The absence of learning for the OFF_ran_ group differs from previous findings^7^. Differences in methodology might explain the discrepancy between results. In the present experiment we used a different velocity profile and peak velocities and the required saccade amplitudes were more variable. This may have increased the difficulty of our task and impaired learning. Furthermore, the actual saccade amplitude was biased towards the average coordinate of the three required saccade amplitudes (i.e., 15°). To verify that saccade averaging was not caused by participants trying to reproduce the same response even though the required saccade changed over trials, we calculated the variability of saccade amplitude between trials. We used a sliding window of 5 trials with an overlap of 2 trials (*inset* 2 C). This analysis revealed a significantly larger inter-trial variability for the OFF_ran_ group than for the OFF group, indicating that participants did not attempt to reproduce the same saccade amplitude across trials (ANOVA, *F*_(1, 20)_ = 4.7, *p* = 0.04). This result implies that participants did comply with the demands of the task.

### Transfer condition: offline task

**Figure 3:**
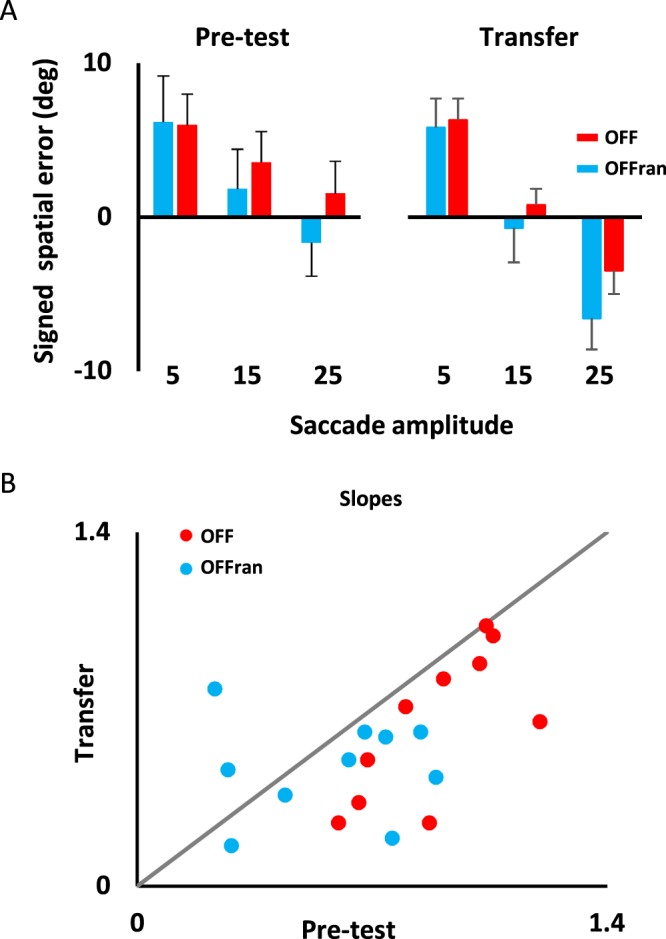
Transfer offline: (**A**) Signed spatial error during the pre-test and the transfer for the three saccade amplitudes. A larger error anisotropy between the targets is depicted during transfer compared to the pre-test. (**B**) Individual slope values during the transfer are plotted against regression slope values during the pre-test. The slope values (blue and red dots) lie below the identity line (gray line) illustrating that the slopes of regression were smaller during the transfer than during the pre-test for both the OFF_ran_ and the OFF group (except for two participants in the OFF_ran_ group).

The different methodologies used during the pre-test (i.e., 3 initial target eccentricities) and practice (i.e., 3 amplitudes of chair rotation) conditions yielded contrasting performance between the OFF and OFF_ran_ groups. The slope of regression between pre-test and practice conditions (0.72 vs. 0.14, respectively) was attenuated for the OFF_ran_ group, revealing a larger anisotropy in saccade amplitude. These results suggest that the underlying mechanisms for the two offline tasks differ. To determine whether any transfer occurred between these tasks, all participants were transferred to a condition identical to the pre-test (i.e., initial targets were presented at 55°, 45° or 35° to the left of the participant’s midline before a 60° body rotation). Figure 3A illustrates the signed spatial error for the pre-test and transfer conditions. A significant condition by saccade amplitude interaction (ANOVA, *F*_(2, 40)_ = 11.2, *p* < 0.001) revealed that the difference in signed spatial error between saccade amplitudes was greater during the transfer than the pre-test. This larger anisotropy during transfer was caused by a decrease in spatial error for the 15° saccade and an increase in spatial error for the 25° saccade. To better illustrate this result, a regression was calculated between the actual and the required saccade for each participant and slope values in the transfer condition were compared to slope values in the pre-test condition. When plotting individual slope values of the transfer against the pre-test (Fig. 3B), all except two participants of both the OFF and the OFF_ran_ groups showed a decrease in their slope during transfer. This can be seen in the scatter plot showing that the slope values lie below the identity line. The mean slope was reduced (*F*_(2, 20)_ = 21.2, *p* = 0.001) from pre-test values of 0.88 ± 0.2 and 0.72 ± 0.3 to transfer values of 0.64 ± 0.3 and 0.48 ± 0.2 for the OFF and the OFF_ran_ groups, respectively. No significant difference between these conditions was found for the intercept (*F*_(1,20)_ = 0.78, *p* = 0.39, not illustrated). The reduction in slope values between pre-test and transfer conditions implies that the larger anisotropy experienced during practice transferred between offline tasks. Specifically, it implies that practicing under the three chair rotations condition rather than the three target eccentricities condition yielded a poorer performance and caused a retrograde interference.

### Weighted optimization estimation

**Figure 4:**
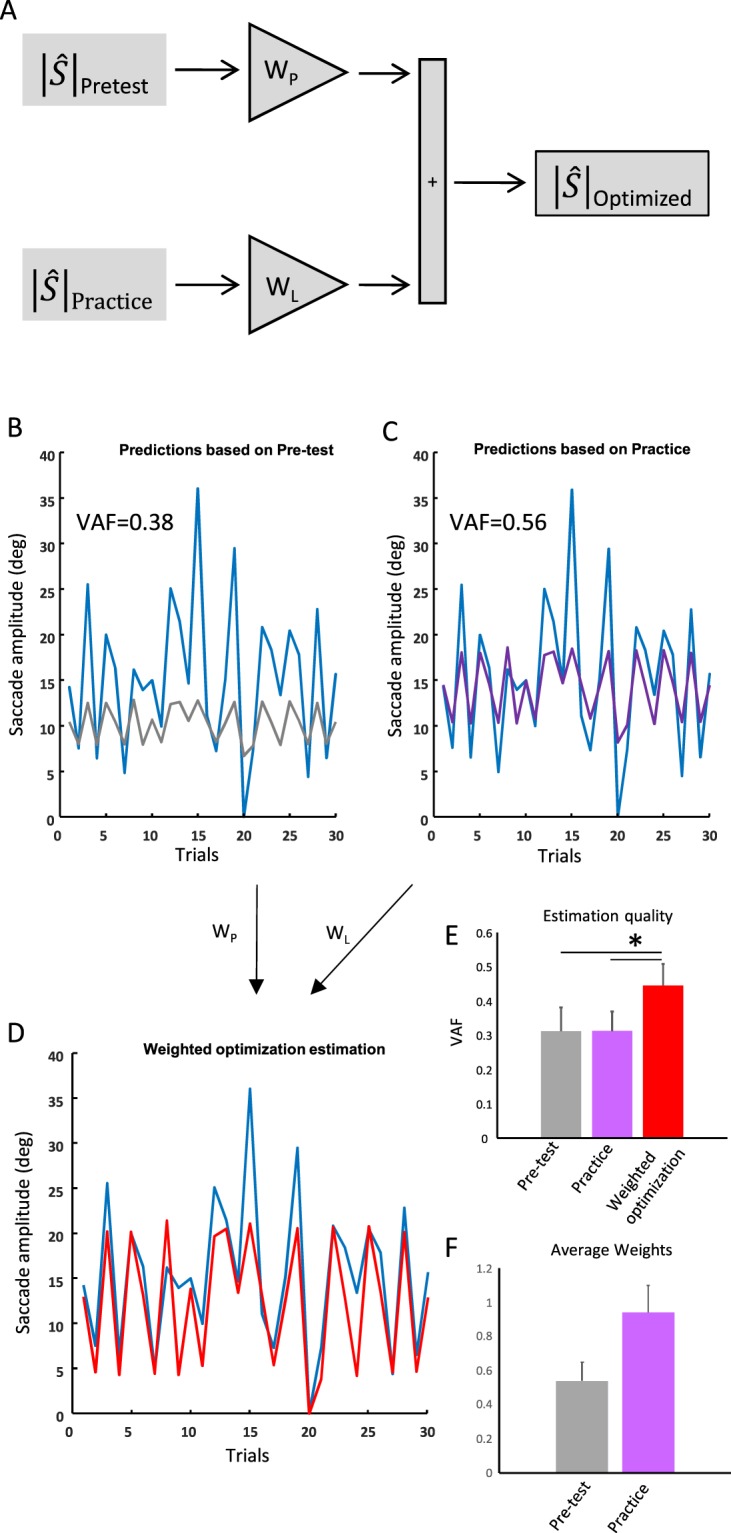
Weighted optimization estimation. (**A**) Schematic representation of the weighted optimization model. In panels (**B**–**D**) the solid blue line represents the saccade amplitude during the transfer condition. (**B**) Saccade amplitude predictions based on the pre-test data at each target eccentricity. The gray solid line represents the prediction based on the pre-test. (**C**) Saccade amplitude predictions based on the practice data at each target eccentricity. The purple line represents the prediction based on the practice. (**D**) Weighted optimization estimation. The red line represents the best estimation using the optimized weights of the pre-test and the practice data. (**E**) Average of the quality of the fitting procedure (VAF) using the linear model with the pre-test data (gray bar), the practice data (purple bar) and the weighted optimization model (red bar). The VAF was significantly larger for the weighted optimization model. (**F**) The gray bar represents the averaged best weight based on the pre-test data. The purple bar represents the averaged best weight based on the data from practice. * represents p < 0.05.

Results of the transfer condition suggest that practice caused retrograde interference between offline tasks. To further evaluate this result, we estimated the contribution of both pre-test and practice conditions on the transfer condition by computing a weighted optimization model (Fig. 4A). First, we assessed whether the pre-test performance could predict responses during the transfer. Saccade amplitudes during transfer were predicted based on a linear model that used the pre-test data as an input (${|\hat{S}|}_{{\rm{pre}} \mbox{-} \mathrm{test}}$). Then, we repeated the same procedure using the practice data as an input to assess whether practice explained the responses during the transfer (${|\hat{S}|}_{{\rm{practice}}}$). To evaluate the performance of the model in estimating the saccade amplitude during transfer, the variance-accounted-for (VAF) was computed:

2 $$VAF=1-[var(\hat{S}-S)/var(S)]$$

where $\hat{S}\,\,$represents the estimated saccade amplitude and *S* the actual saccade amplitude. A VAF of 1 indicates a perfect fit to the data. Note that the VAF in such a linear model is equivalent to the square of the correlation coefficient (${R}^{2}$). As detailed in the previous section, the linear model typically underestimated responses during the transfer (see Fig. 3). Next, we characterized the responses using a “weighted summation model”. We estimated the weights for pre-test (W_p_) and practice (W_L_) conditions to give the best fit to the transfer data (${|\hat{S}|}_{Optimized}$).

3 $${|\hat{S}|}_{Optimized}={w}_{p}{|\hat{S}|}_{{\rm{Pre}}-{\rm{test}}}+{w}_{L}{|\hat{S}|}_{{\rm{Practice}}}$$

Figure 4(B–D) presents the saccade amplitude during the transfer (i.e., blue traces) and the model estimations (i.e., gray and purple traces) when considering: (B) pre-test condition, (C) practice condition and, (D) the weighted combination of the pre-test and practice conditions, for a representative participant. The saccade amplitude was better estimated by the weighted sum of the pre-test and practice (i.e., Fig. 4D) rather than these conditions taken alone (i.e., Fig. 4B,C). Across participants, the VAF was significantly better (*F*_(2,18)_ = 4.8, *p* = 0.03) for the weighted combination of the pre-test and practice condition (Fig. 4E). We found that the optimized weight was slightly larger (Fig. 4F) for practice (W_L_) than for the pre-test (W_p_). This difference, however, did not reach statistical significance (*p = *0.09). Overall, these results suggest that the mechanisms involved in updating the position of a single target for three chair rotation amplitudes transferred to the condition with three target eccentricities.

### Online processing of vestibular information

**Figure 5:**
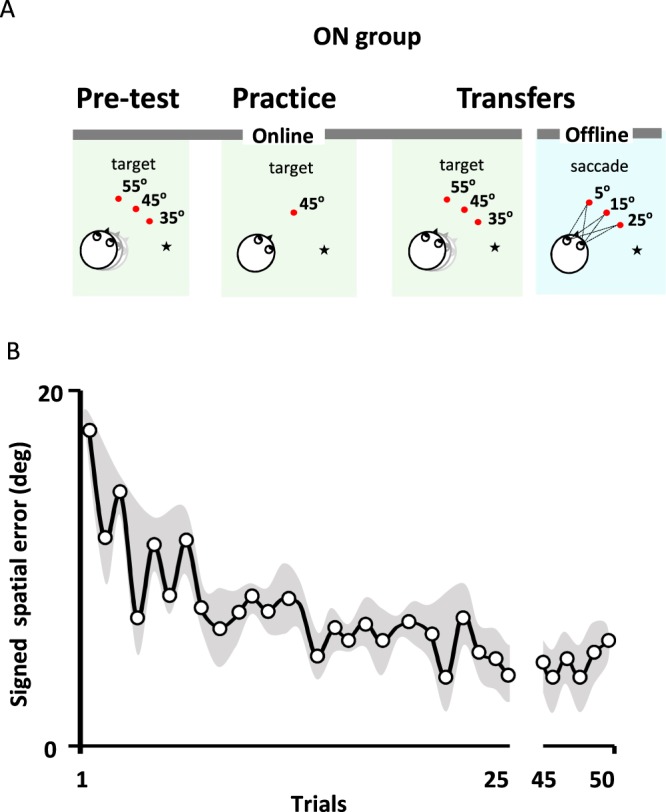
(**A**) Online procedure description: The star shows the starting position before rotation and the red dots illustrate the positions of the initial targets. Pre-test: Participants pressed a push-button when they perceived that their body’s midline had crossed the memorized position of one of the three initial target positions (55°, 45° or 35°) during the ongoing rotation. Practice: In every trial, the initial target position was at 45° to the left of the starting position. Transfers: Participants reproduced the task they did during pre-test (online task, shaded green box). Thereafter, participants performed the transfer in the offline condition (shaded blue box). (**B**) Learning during online practice. Signed spatial error over 50 trials (solid black line). The signed spatial error is largely reduced after the first trial. A slighter reduction of the error is observed during the remaining trials. The gray shading represents the standard deviation.

We observed that practice resulted in retrograde interference between offline tasks. To find stronger evidence in favour of a generalization mechanism resulting from learning, we selected an online spatial updating task for which learning occurs in the presence of external feedback^11,12^. The initial targets were to the left of the participant’s midline before body rotation (see Fig. 5A). The magnitude of chair rotation was 70°, 80° or 90° and peak angular velocities were 83°/s ± 4°/s, 88°/s ± 4°/s and 92°/s ± 3°/s, respectively. During the rotation, participants were asked to respond, by using a push button, when they perceived that their midline crossed the target position.

### Practice condition: online task

During practice, initial target was located 45° to the left of the participant’s midline. The ON group showed a rapid reduction of the signed spatial error within the first trials (Fig. 5B). Thereafter, a slight decrease in spatial error was observed through the remaining trials. Reduction of the signed spatial error was supported by a significant main effect of the Epochs (3 first trials vs. 3 last trials), ANOVA, *F*_(1,9)_ = 32.1, *p* = 0.000. We have characterized the learning time constant by fitting a falling exponential curve on the signed spatial error (see Equation (1)). The median time constant of learning was $\tau =5.5$trials (nonlinear square fit, median R^2^ = 0.4).

### Transfer between online and offline tasks

**Figure 6:**
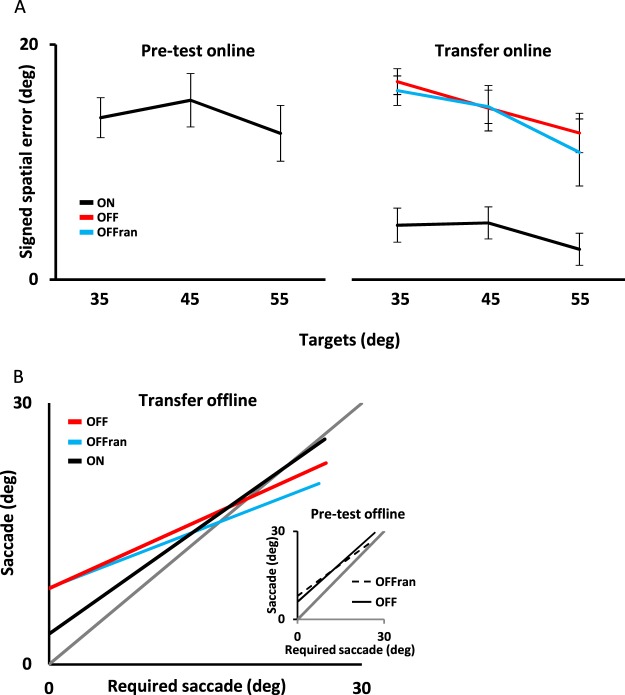
Transfer within and between the tasks. (**A**) Online spatial error during the pre-test (black line: left panel) and after training (black line: right panel). Training with the 45° target improved performance on the 35° and 55° targets. However, offline training did not improve performance when tested during the online transfer (solid red line and solid blue line, respectively). (**B**) Mean regression lines between expected and actual saccades during the offline transfer condition for the ON (black solid line), OFF (red solid line) and OFFrand (blue solid line) tasks. No significant differences were found between the regression line for the ON task during the transfer condition and the regression lines for the OFF and OFF_ran_ tasks during the pre-test condition (inset, solid black line and dashed line, respectively).

Performance on the practice condition improved for the online task, conversely to the offline task. This improvement implies that the brain learned to use vestibular signals to estimate its spatial position. To test whether such learning is specific to the context, participants performed two transfer conditions. First, we tested whether learning transferred toward multiple target positions. To this end, the performance of the transfer online was compared with that of the pre-test online conditions, where three targets position were presented pseudo-randomly at 35°, 45° or 55°. As illustrated in Fig. 6A (black lines), learning to update a 45° target during body rotation resulted in a significant reduction in the signed spatial error for all three target eccentricities, ANOVA main effect of conditions (pre-test vs. transfer), *F*_(1,18)_ = 37.4, *p* = 0.000. This result replicates previous findings^12^ and strengthens the view that vestibular signals can be used to keep a stable representation of various elements in spatial memory when being trained towards a single target position. Second, we tested whether the mechanisms involved during offline and online practice (i.e., enhanced anisotropy or learning) transferred from one task to the other. The panel to the right in Fig. 6A illustrates the signed spatial error when performing the online task. As depicted, the signed spatial errors for the OFF and the OFF_ran_ groups (coloured lines) were similar to those observed for the ON group during the pre-test, ANOVA main effect of groups, *F*_(2,26)_ = 0.64, *p* = 0.94. As well, no significant interaction between groups and targets was revealed (ANOVA, *F*_(4,52)_ = 1.5, *p* = 0.22) suggesting that the larger anisotropy observed for the OFF groups during the practice condition did not transfer to the online task. Similarly, we tested whether learning online spatial updating would enhance performance of the offline task. Figure 6B illustrates the mean regression line for all groups during the transfer offline condition. During the offline transfer condition, for the OFF, OFF_ran_ and ON groups, the mean regression lines had slopes of 0.64 ± 0.3 (R^2^ = 0.60), 0.48 ± 0.2 (R^2^ = 0.45) and 0.81 ± 0.3 (R^2^ = 0.65), and intercepts of 7.4° ± 4.3°, 9° ± 6.4° and 1.8° ± 3.8° (mean ± SD), respectively. No significant difference was found between groups when comparing slope values (one-way ANOVA, *F*_(2, 28)_ = 2.9, *p* = 0.07). Conversely, the intercept was lower for the ON group than for the OFF and OFF_ran_ groups, whereas between the OFF groups it did not differ (one-way ANOVA, *F*_(2, 28)_ = 5.9, *p* = 0.008). However, when comparing the results of the ON group during transfer with those of the OFF groups during the pre-test (Fig. 6B *inset*), no significant difference was found between the slopes (one-way ANOVA: *F*_(2.28)_ = 1.1, *p* = 0.36) and intercepts (one-way ANOVA: *F*_(2.28)_ = 1.9, *p* = 0.16) calculated for each condition. This result suggests that the improvement in performance during online practice did not transfer to the offline task.

## Discussion

Spatial updating preserves the capacity of our brain to maintain perceptual stability as we move, allowing us to properly interact with our environment. In the absence of visual information and efferent copy signals, however, the brain likely changes its sensory cues to maintain optimized spatial updating. Because vestibular signals encode self-motion^4^, optimized spatial updating can be achieved using vestibular signals. The extent to which this mechanism generalizes across tasks is crucial information for determining its functional role in motor and perceptual behaviors. To address this question, we used a learning transfer paradigm to determine whether improvement in spatial updating would transfer across the workspace and between spatial updating contexts. Results revealed that vestibular learning does transfer across the workspace but does not transfer between different spatial updating contexts. These results offer important insight into mechanisms that use vestibular input for spatial updating and suggest that the type of processing performed during learning could constrain task generalization.

The ability of the brain to change its sensory input to perform a task has been primarily evidenced in congenitally sensory deprived individuals. Such plasticity has traditionally been the hallmark of sensory loss^19,23^. Recent observations, however, suggest that pre-existing neural networks for a non-dominant input in specialized areas can be unmasked to enhance perceptual experience or can substitute for the temporary absence of a dominant sensory input without sensory loss^21,22,24^ (reviewed in^18,25^). However, the precise nature of such sensory change remains unclear. For instance, unimodal functions such as colour recognition are unlikely to use a sense other than vision^21,26^ while supra-modal functions (i.e., function that are shared across senses, such as stimulus localization) have been found to promote the use of a variety of sensory signals. In this regard, it is suggested that a brain region can preserve a supra-modal function by changing its sensory source^27^. We propose that learning to use the vestibular signal allows the brain to preserve its ability to update the visual field in the absence of other sources of information, and that this learning will generalize between tasks that engage similar mechanisms. Here we show that spatial updating is generalizable within a given context. For the online task, this was demonstrated by the improvement of performance between the pre-test and online transfer condition and for the offline task, it was revealed by retrograde interference across the workspace. Specifically, saccade amplitude showed large anisotropy and averaging following offline practice. One possibility is that saccade averaging was implemented during practice and persisted in oculomotor memory^28–31^ when transferred toward multiple initial targets. However, using a similar task (i.e., one target position that had to be updated following three possible body rotation amplitudes), Israel *et al*.^7^ reported that saccade response accuracy improved with practice rather than averaging. It is likely that the saccade averaging observed in the present study is an epiphenomenon resulting from methodological differences. In the present study, the variability in the amplitude of body rotation presumably added noise in the vestibular integration. Although the position discrimination threshold resulting from vestibular integration remained unknown, it is possible that this noise decreased the ability to make the distinction between the different amplitudes of rotation and thus decreased the precision of vestibular spatial updating. Further, vestibular heading discrimination thresholds were found to vary between 5° and 15° ^32^ for translational motions. In the present study, the amplitudes of rotation differed by 10° (as in^7^) which lies within the values where the precision is reduced, resulting in perceptual averaging. Further studies are needed to address this important question. Most importantly, changes in vestibular processing that took place during offline practice did not generalize to online vestibular processing.

Generalization was not found between online and offline tasks. This may be due to the different perceptual biases experienced during whole-body rotation. In a condition of symmetrical and high acceleration/deceleration phase (i.e., a condition like our rotation profile), angular motion was overestimated during the acceleration phase (the gain between actual and perceived displacement was 1.19) and underestimated during the deceleration phase (gain: 0.67^33^; see also^34^ for similar results). However, the average gain was close to 1 (gain: 0.99) after the rotation stopped, suggesting an accurate perception of the total angular displacement. Because spatial updating relies on perception of body rotation, any perceptual bias experienced during or after the rotation will induce error when remapping the target position. Accordingly, our results showed that participants from the ON group produced larger overestimation errors than the participants in the OFF groups (i.e., pre-test). This could be accounted for by the higher perceptual gain experienced during the acceleration phase, when participants planned their response^11^. Further, practicing the online task likely permitted subjects to overcome their perceptual bias and reduce their spatial error. It is most likely that participants in the OFF groups also experienced variation in their motion perception during the rotation, but as this perceptual bias vanished at the end of the rotation, practicing the offline task did not teach them to reduce this bias. Therefore, the OFF groups showed large overestimation errors when transferred to the online task. This suggests that the perceptual error experienced during practice could determine generalization.

Furthermore, spatial updating errors do not arise exclusively from misperception of body rotation, but also from the transformation of a vestibular signal into a retinal signal when updating the retinotopic map^5^. Some partial transfer should be observed between the two spatial updating tasks if the mechanism used to remap the position of the targets is similar. The absence of such a partial transfer suggests that offline and online spatial updating rely on independent transformation processes. In the present experiment, vestibular-derived self-motion can be processed with disregard to the visual field^35^ and stored in spatial memory^36,37^ when performing the offline task. To attend to the visual field, one may reconstruct or infer the position of the visual stimuli at the end of the motion. Interestingly, it has been found that loss of vestibular input impairs spatial memory in hippocampal formation^38–40^. Such a delayed transformation process, however, cannot be used during online spatial updating because of the time constraint. Rather, to perform the online task, a continuous transformation between the vestibular signal and the target position is necessary, implying minimal contribution of spatial memory^36,41^. The lack of transfer between online and offline spatial updating tasks can be explained by a difference in the brain structures involved during the transformation process^42^.

The first stage in processing vestibular signals involves the same class of neurons (i.e., vestibular-only neurons). Thus, any difference in integration of vestibular signals for spatial perception and updating would likely occur at a supra-thalamic level. In the present experiment, it is noteworthy that the pursuit system is used to cancel the vestibulo-ocular reflex (VOR) during whole body rotation^43^. During VOR cancellation, for which eye velocities and target retinal velocity are null, the neurons in the pursuit network remain sensitive to gaze velocity^42^ which can also be used as an input signal for spatial updating. Moreover, because the pursuit system can compute predictive mechanisms to overcome neural delay^44–47^, it represents a suitable candidate to contribute to online spatial updating. Further, the temporo-parietal junction is known to be involved in the integration of vestibular signals for offline spatial updating (see^48^ for a review). Thus, the contribution of different neural networks could explain the lack of generalization between online and offline spatial updating. Alternatively, online and offline spatial updating mechanisms may be computed in the same cortical area that uses multiple reference frames^49,50^. For instance, Tramper and Medendorp^51^ tested spatial representations of visual space during motion and found that the brain uses a weighted combination of eye-centered and body-centered frames of reference. Thus, the lack of generalization could result from the brain using different reference frames, or a weighted combination, to perform online and offline spatial updating tasks. While not mutually exclusive, future studies will be needed to investigate what prevents generalization (i.e., the neural network or reference frame) at the cortical level.

## Conclusion

We have demonstrated that within a given context, spatial updating performance transfers from trained to untrained targets. Spatial updating, however, does not generalize across different contexts (i.e., offline to online spatial updating and vice versa), emphasizing that the mechanisms that process and transform vestibular signals operate differently during learning. Results of this behavioural study contribute to our understanding of the mechanism involved in spatial updating and pave the way for future neurophysiological studies aiming at investigating cortical mechanisms leading to vestibular spatial learning.

## Method

### Participants

Thirty healthy individuals (including 12 females) between 20 and 30 years of age participated in this study. All individuals reported normal or corrected-to-normal vision and no history of motor or sensory impairments. The experimental protocol was approved by Laval University Biomedical Ethics Committee. All participants gave written informed consent according to Laval University Biomedical Ethics Committee guidelines and the study was conducted in accordance with the Declaration of Helsinki.

### Tasks and apparatus

The tasks and apparatus were modeled on previous published studies from our laboratory^11,12^. Participants were seated on a rotating chair facing a semicircular panel with a radius of 1.5 m in a completely dark room (Fig. 1A). They were secured to the rotating chair with a 4-point belt system and chin support that prevented head-on-body movements during passive body rotation. To attenuate the vestibulo-ocular reflex (VOR) during passive body rotation, participants were instructed to maintain their gaze at a chair-fixed target (i.e., a red light-emitting diode affixed to the chair) located straight ahead at eye level. Note that the use of a chin rest and a chair-fixed target attenuated neck and eye muscle proprioceptive information as sources of information for spatial updating. At the beginning of each trial, participants were first placed in the starting position (represented by the star in Fig. 1A) and asked to gaze at the fixation point (i.e., the red light-emitting diode) located straight ahead on the semicircular panel at eye level. This was designed to ensure that the initial target position appeared at the same retinal location for a given eccentricity. Then, the target was illuminated for 1 s (the red light-emitting diode on the semicircular panel). Participants were asked to locate and memorize the position of the target without making a saccade. Thereafter, the target was extinguished, and participants were rotated around the vertical axis (Fig. 1A, middle panel). All rotations were counter-clockwise. The magnitude of chair rotation was 60°, 70°, 80° or 90° of the amplitude depending on conditions (see Procedure section below). The chair was rotated following a bell-shaped velocity profile, as this simulates the velocity profiles of natural head movements^52^. Peak angular velocities were scaled according to the amplitude of chair rotation, with means of 73°/s ± 3°/s, 83°/s ± 4°/s, 88°/s ± 4°/s and 92°/s ± 3°/s, for the 60°, 70°, 80° or 90° rotations, respectively, and chair rotation lasted approximately 1.5 s (Fig. 1A *inset*). After completion of the trial, participants were returned to the starting position for the next trial. Chair angular position was measured with an optical encoder (Model H5S, US Digital, Vancouver, WA, USA) fixed at the chair’s center of rotation and monitored at 1000 Hz with a 16-bit A/D board (Model AT-MIO-16DE-10, National Instruments Corporation, Austin, TX, USA). During rotation (i.e., the online task, see Procedure section below), participants’ responses were measured using a push-button that produced an analog signal, recorded synchronously with the angular position of the chair. When the response was given after the rotation (i.e., the offline task, see Procedure section below), participants were asked to gaze at the new position of the memorized target and responses were measured by recording horizontal eye movements using electrooculography (Biomedica Mangoni, model BM623, Pisa, Italy).

### Procedure

Participants were assigned randomly to one of three experimental groups (n = 10, respectively). For the ON group, participants were trained to press a push-button when they perceived that their body’s midline had crossed the memorized target during the ongoing body rotation (online task)^11,12^. For the second task, there were two groups. Participants were trained to produce saccadic eye movements to the memorized target after their body rotation had stopped and the chair-fixed target was switched off (offline task)^7–10^. For one group, the amplitude of body rotation was constant (OFF group) while it varied for the other group (OFF_ran_ group). All participants took part in four experimental conditions: pre-test, practice, transfer to other target eccentricities, and transfer to the unpracticed task.

### Procedure for OFF groups

Pre-test: During the pre-test, all participants performed 1 block of 15 trials with no feedback and with a chair rotation amplitude of 60°. Initial targets were presented pseudo-randomly at 35°, 45° and 55° to the left of the subject’s midline with the restriction that every target was presented equally within the pre-test condition (i.e., 5 trials per target). Following passive body rotation, the new position of the memorized targets relative to each participant’s midline was precisely calculated as the difference between the chair position and target position. Accordingly, participants were required to produce saccade amplitudes of 25° ± 0.8°, 15° ± 0.7° or 5° ± 0.8° to gaze accurately at the memorized targets (Fig. 1B, left Pre-test panel).

Practice: During the practice offline condition, for all 51 trials, the initial target was always located at 45° from the starting position. For the OFF group, the chair rotation magnitude was 60° on every trial. Participants had to produce a saccade amplitude of 15° ± 0.7° to gaze accurately at the memorized target (Fig. 1B, middle Practice panel). Because the amplitude of the required saccade was fixed on every trial, the oculomotor system could learn to produce a 15° saccade regardless of the vestibular information. To control for that possibility, for the participants in the OFF_ran_ group, the initial target was presented at 45° to the left of the subject’s midline but the chair rotation amplitude varied pseudo-randomly across trials (i.e., 70°, 60° and 50°, Fig. 1B, middle Practice panel). According to the chair rotation amplitude, participants were required to produce saccade amplitudes of 25° ± 0.7°, 15° ± 0.7° and 5° ± 0.7° to gaze accurately at the memorized target. Thus, participants needed to process vestibular information. At the end of the trial, when eye position had stabilized at the perceived target position, the target was lit up to provide visual feedback. If a perceptual error was made, participants were instructed to refrain from performing a corrective saccade to the target. The electro-oculogram (EOG) traces revealed that participants complied with this instruction (Supp. Fig. S1A, gray line).

Transfers: Following practice, participants performed two transfer conditions (Fig. 1B, right Transfers panel). Participants first performed the transfer offline condition, which was identical to the pre-test offline condition with the exception that 10 trials per target were performed. Thereafter, to assess if learning the offline task had transferred to the online task (i.e., the transfer online condition), participants performed the transfer online condition (see next paragraph).

### Procedure ON group

Pre-test: In the pre-test online condition, participants performed 1 block of 15 trials with no feedback and a chair rotation amplitude of 80°. Initial targets were presented pseudo-randomly at 35°, 45° and 55° to the left of the participant’s midline (i.e., 5 trials per target, Fig. 5A, left Pre-test panel).

Practice: During the practice online block (51 trials), the initial target was located at 45° to the left of the participant’s body midline (Fig. 5A, middle Practice panel). Visual feedback was provided on each trial; the target lit up simultaneously with the subject’s response. During practice, three different chair rotation amplitudes (i.e., 70°, 80° and 90°) were selected pseudo-randomly adding variability in the instance that participants crossed the target. This procedure was mandatory in order to prevent learning of internal timing during training^11^. Notably, because the temporal occurrence of the target followed a normal distribution (Supp. Fig. S1B), participants could have learned the mean temporal occurrence to predict the location of the target (Bayesian theory^53^). To verify for this alternative, the participant’s response was subtracted from the mean temporal occurrence of the target (i.e., temporal error). As shown in Supplementary Fig. S1C, no reduction in temporal error was noted across practice.

Transfers: Following practice, participants performed two transfer conditions (Fig. 5A right Transfers panels). Participants first performed the transfer online condition which was identical to the pre-test condition with the exception that 10 trials were performed for each target. Then, participants performed the transfer offline condition (30 trials).

### Data analysis

For the offline task, saccade amplitude was measured from the calibrated EOG signal and calculated as the difference between the value of the EOG signal at the beginning and at the end of the saccade. Saccade onset and offset were defined as the mean value of the EOG signal comprised within the 250 ms preceding or following the peaks of the second derivative (Supp. Fig. S1D). Signed spatial error was calculated as the difference between the amplitude of the saccade response and the amplitude of saccade required to gaze accurately at the target. Undershoots were assigned negative value while overshoots were assigned positive value. For the online task, the signed spatial error was calculated from the difference in degrees between the target angular position and the chair position at participant’s response. Clockwise errors (i.e., when participants overestimated their body rotation) were assigned positive value, while counter-clockwise errors were assigned negative value (i.e., when participants underestimated their body rotation). The absolute spatial error value was also calculated. In cases where spatial error decreased with practice trials, the learning rate across trials was characterized by fitting an exponential model to the learning curve of each participant:

1 $${\rm{SE}}(i)={\rm{\alpha }}+{{\rm{\beta }}}^{-(i/t)}$$

where SE is the spatial error on trial (*i*), α is performance reached at the learning plateau, β is the gain and *τ* is the time constant of learning.

### Statistics

Parametric statistics (ANOVA) and mean values were considered when the data were distributed normally. Greenhouse-Geisser corrections were applied when epsilon values were smaller than 1. All significant main effects involving more than two means were broken down according to Dunn’s technique. Significant interactions were broken down by computing simple main effects, followed by post hoc comparisons when there were more than two means. To determine the correlation between the amplitude of the required saccade and the saccade produced by the subject, linear regression analyses were computed. All effects are reported at p < 0.05 (adjusted for the number of comparisons). When data deviated from the normal distribution, Mann-Whitney-Wilcoxon (MWW) non-parametric analysis was performed.

## Supplementary information

Supplementary figure

## Data Availability

The datasets generated during and/or analysed during the current study are available from the first author on reasonable request.
