# Supplementary material for: Learning to use vestibular sense for spatial updating is context dependent
Source: Sci Rep. 2019 Aug 1;9:11154. doi: 10.1038/s41598-019-47675-7 (PMC6671975; doi:10.1038/s41598-019-47675-7)
Supplement: Supplementary file 1 — Supplementary figure [file 41598_2019_47675_MOESM1_ESM.docx]

The vestibular sense and learning how to use it: What governs generalization?

Isabelle Mackrous^1^, Jérôme Carriot^1^ and Martin Simoneau^2,3*^

^1^Department of Physiology, McGill University, Montreal, QC, Canada; ^2^Centre Interdisciplinaire de Recherche en Réadaptation et Intégration Sociale (CIRRIS), Québec, QC, Canada; ^3^Département de kinésiologie, Faculté de médecine, Université Laval, Québec, QC, Canada

**
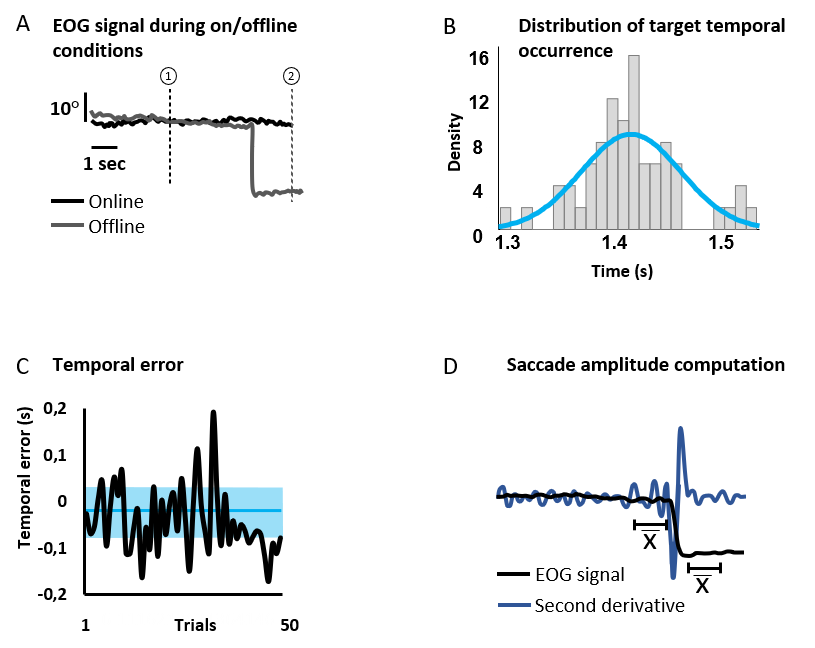
**

**Supplementary Figure S1:** A) EOG signal during online (black trace) and offline (gray trace) trials. At the target presentation, participants did not saccade toward the target but maintained fixation straight ahead. As instructed, participants did not saccade to the target when feedback was provided during (1) or after the chair rotation (2). B) During the rotation, the moment at which the participant crossed the position of the target followed a normal distribution (blue line). C) The temporal error (black line) did not diminish during practice. The blue line and shaded area represent the mean temporal occurrence and standard deviation, respectively. D) Saccade amplitude calculation. To identify the onset and offset of the saccade, we computed the second derivative of the EOG signal (blue trace). Saccade onset was defined as the mean value of the EOG signal (black trace) within the 250 ms preceding the first main peak of the second derivative. Saccade offset was defined as the mean value of the EOG signal within the 250 ms following the second main peak of the derivative.
